# Prevention and risk reduction in Canadian dementia strategies: a scoping review

**DOI:** 10.1002/alz70860_102298

**Published:** 2025-12-23

**Authors:** Lindsay MK Wallace, Sebastian Walsh, Carol Brayne

**Affiliations:** ^1^ Dalhousie University, Halifax, NS, Canada; ^2^ Cambridge Public Health, University of Cambridge, Cambridge, Cambridgeshire, United Kingdom

## Abstract

**Introduction:**

Evidence suggests that up to 40% of dementia risk is modifiable (Livingston et al., 2020, *Lancet*). However, the majority of interventional evidence for dementia risk reduction relies on individual behaviour change, which does not account for social context and is unlikely to produce benefit at scale (Walsh et al., 2022, *Lancet Healthy Longevity)*. Canada has been a leader in dementia research globally but has lagged in policy supporting dementia prevention and risk reduction. Our objective was to review existing dementia strategies and policies to assess their focus on population‐level prevention and risk reduction in the Canadian context.

**Methods:**

Primary research databases and grey literature (PubMed, healthevidence.org, customized google search databases) were searched using terms related to “dementia”, “policy”, and ”province/territory“. Results were independently double‐screened, and included if they were active, finalised policy documents, written in French or English, explicitly focused on dementia from a Canadian provincial or federal authority. Data were extracted based on directed content analysis into pre‐determined categories that were iteratively updated: 1) priorities/actions/interventions addressing prevention or risk reduction; 2) Equity and inclusion; 3) Implementation and evaluation.

**Results:**

Seven of thirteen provinces/territories had published dementia strategies, in addition to a national strategy. Only half included any risk reduction/prevention in their priorities, all of which were public awareness raising activities. People living with dementia and their caregivers were included in 5/8 strategies and few provinces had an implementation plan or dedicated funding.

**Conclusion:**

Canadian dementia policy does not prioritize prevention, particularly risk reduction strategies that are population‐based and stand to create the most impact. Future dementia strategies should focus on population‐level prevention and risk reduction as a key priority in order to produce large, equitable reductions in dementia prevalence. Further, input from people with lived experience should be integral to the development of new policies, as well as built‐in organizational accountability and funding to ensure timely uptake and success of policy objectives.

